# Ocean-Scale Patterns in Community Respiration Rates along Continuous Transects across the Pacific Ocean

**DOI:** 10.1371/journal.pone.0099821

**Published:** 2014-07-21

**Authors:** Jesse M. Wilson, Rodney Severson, J. Michael Beman

**Affiliations:** Life and Environmental Sciences, Environmental Systems, and Sierra Nevada Research Institute, University of California Merced, Merced, California, United States of America; Argonne National Laboratory, United States of America

## Abstract

Community respiration (CR) of organic material to carbon dioxide plays a fundamental role in ecosystems and ocean biogeochemical cycles, as it dictates the amount of production available to higher trophic levels and for export to the deep ocean. Yet how CR varies across large oceanographic gradients is not well-known: CR is measured infrequently and cannot be easily sensed from space. We used continuous oxygen measurements collected by autonomous gliders to quantify surface CR rates across the Pacific Ocean. CR rates were calculated from changes in apparent oxygen utilization and six different estimates of oxygen flux based on wind speed. CR showed substantial spatial variation: rates were lowest in ocean gyres (mean of 6.93 mmol m^−3^ d^−1^±8.0 mmol m^−3^ d^−1^ standard deviation in the North Pacific Subtropical Gyre) and were more rapid and more variable near the equator (8.69 mmol m^−3^ d^−1^±7.32 mmol m^−3^ d^−1^ between 10°N and 10°S) and near shore (e.g., 5.62 mmol m^−3^ d^−1^±45.6 mmol m^−3^ d^−1^ between the coast of California and 124°W, and 17.0 mmol m^−3^ d^−1^±13.9 mmol m^−3^ d^−1^ between 156°E and the Australian coast). We examined how CR varied with coincident measurements of temperature, turbidity, and chlorophyll concentrations (a proxy for phytoplankton biomass), and found that CR was weakly related to different explanatory variables across the Pacific, but more strongly related to particular variables in different biogeographical areas. Our results indicate that CR is not a simple linear function of chlorophyll or temperature, and that at the scale of the Pacific, the coupling between primary production, ocean warming, and CR is complex and variable. We suggest that this stems from substantial spatial variation in CR captured by high-resolution autonomous measurements.

## Introduction

Microorganisms have profound effects on their surrounding environment, chemically modifying habitat and affecting other organisms through their biogeochemical activity [1], [2], [3]. Some of the most fundamental metabolic functions—in terms of both cell function and environmental importance—involve the production and consumption of oxygen. Oxygenic photosynthesis and aerobic respiration are basic measures of ecosystem function because the production, respiration, cycling, and overall availability of carbon affect everything from the number of trophic levels, to the types of organisms present [4]. Oxygenic photosynthesis dictates the amount of oxygen available for aerobic organisms (including both microbes and larger organisms), and photosynthesis in the ocean is almost exclusively microbial, accounting for approximately half of global oxygenic photosynthesis [5]. The subsequent oxidation of organic carbon with oxygen yields tremendous amounts of free energy, making aerobic respiration a preferred method of obtaining energy, and an indication of the total amount of production and activity in an ecosystem.

In many open ocean systems, photosynthesis and aerobic respiration rates tend to be tightly coupled and close to balanced [6], [7], [8], [9], [10], [11]. However, the biological carbon pump depends in part on decoupling between photosynthesis and the respiration of organic carbon, leading to net export of carbon in areas where gross primary production (GPP) ultimately exceeds community respiration (CR). Importantly, GPP and CR are expected to differ in their sensitivity to ocean warming: metabolic theory predicts that both CR and GPP will increase as global temperatures rise [12], but that CR should increase more rapidly than GPP [13], [14], [15], [16], [17], [18]. This scenario leads to a positive feedback for climate change, as a larger fraction of primary production would be channeled through the microbial food web and respired to carbon dioxide [13], [16], [17]. Because this has global implications for marine ecosystems and biogeochemical cycles, understanding how CR is affected by temperature and other environmental variables is critical for our understanding of oceanic carbon cycling.

Recent results indicate that CR does scale with temperature, but the majority of these measurements have been collected in the Mediterranean Sea, and Arctic, Atlantic, Indian, and Southern Oceans [18], [19]. Most of the limited Pacific Ocean measurements are restricted to the vicinity of station ALOHA north of the Hawaiian Islands [20], [21], [22], [23], [24]. While these data are a powerful tool for understanding open ocean processes in the North Pacific Subtropical Gyre (NPSG), microbial community composition and nutrient and carbon sources differ significantly between different open-ocean and nearshore sites, such that relationships between GPP and CR are inconstant [20]. The Pacific also covers a substantially greater area than the Atlantic, and CR in the Pacific Ocean consequently may have a greater influence on global carbon cycling. However, the general lack of information regarding variations in CR across the Pacific means that this is essentially unknown. Of particular interest is how CR varies with natural oceanographic features (like the California Current and Equatorial Upwelling) and proxies for production (such as chlorophyll *a*) compared with temperature-driven changes in CR; understanding and quantifying this variability will lead to a better understanding of the factors driving CR in different regions of the ocean [15], and how they may change. Finally, most of our understanding of large-scale patterns in CR is drawn from syntheses of existing datasets (e.g., [19])—systematic investigations of CR are extremely rare.

We used data continuously collected by autonomous Wave Gliders™ [25] to assess how CR changes according to latitude, temperature, phytoplankton biomass (chlorophyll a), and turbidity across the Pacific Ocean. Quantifying how CR varies across Earth's largest ocean will further our understanding of how climate change may affect CR and ocean carbon cycling in the near future, and we focus on CR alone because of the general disagreement concerning the ideal method for also calculating GPP and net community production (NCP; see below). Our approach dramatically expands the number of CR measurements from the Pacific, and our findings indicate that CR rates are driven by different processes in different regions of the Pacific Ocean.

## Materials and Methods

### Autonomous sampling and data

**Figure 1:**
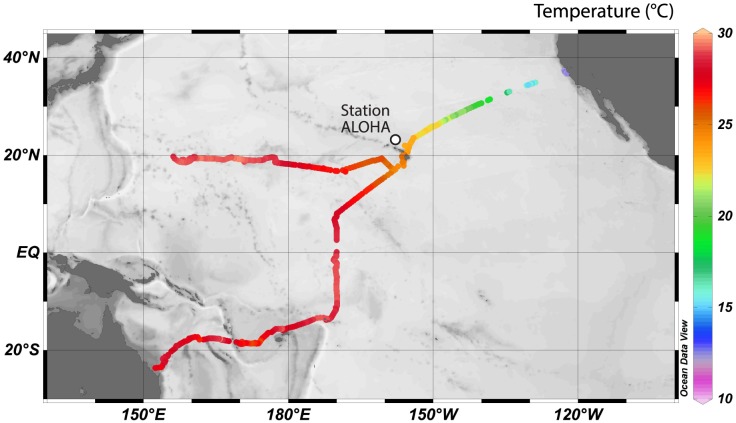
Map of the transpacific paths of the California to Australia and the California to Japan gliders and sea surface temperature as measured by the gliders. Eleven datapoints are not displayed due to obvious GPS errors. Ocean Data View [59] was used to plot and visualize data across the Pacific.

The *Piccard Maru* and *Benjamin* Wave Gliders were deployed by Liquid Robotics [25] on November 17^th^, 2011 off the coast of San Francisco, California as part of the Pacific Crossing or ‘PacX.’ *Benjamin* traveled to Hawaii and turned south, crossing the equator on August 3^rd^, 2012 and arriving in Brisbane, Australia on February 14^th^, 2013 (Figure 1). *Piccard Maru* also traveled to Hawaii and then continued west towards Japan. The glider did not complete the journey to Japan and contact was lost in the Western Pacific. These two datasets are the most spatially- and temporally-extensive of the datasets collected during PacX.

Conductivity, dissolved oxygen concentration (mL L^−1^), oxygen solubility (mL L^−1^), salinity (psu), water temperature (°C), air temperature (°C), and average wind speed (knots), chlorophyll *a* (mg m^−3^), and turbidity (NTU) were recorded by the wave gliders using a Glider Payload CTD, SBE 43F DO sensor, PB200 WeatherStation and C3 submersible fluorometer; data were obtained from the PacX data retrieval site (http://data.liquidr.com/fetch/). Oxygen, salinity, and water temperature were measured by the CTD at 10 seconds intervals, while chlorophyll and turbidity were measured every 2 minutes, and wind speed and air temperature were measured every 10 minutes. Underwater data were collected just below the sea surface (∼0.2 m) while wind speed was measured 1 m above the sea surface. Only data from days when both CTD and weather data are available were used when calculating CR, such that the flux of oxygen from the atmosphere to the ocean or from the ocean to the atmosphere could be accounted for based on wind speed.

### Respiration rate calculations

To quantify respiration rates, we adapted the approach of Needoba, Peterson, and Johnson [26] and calculated (1) the nighttime drawdown of oxygen, as well as (2) the expected flux of oxygen into or out of the mixed layer. We directly followed the Needoba et al. [26] approach for one set of calculations; in these calculations, ‘biological demand of oxygen’ (BDO) [26] is calculated as the difference in oxygen concentrations between any two time points, and then all of these BDO values are summed over each dark period. We performed additional calculations where we modified this approach in two ways. First, in addition to summing changes in dissolved oxygen (DO) over time, we calculated nighttime drawdown of DO based on regressions between apparent oxygen utilization (AOU) and time (AOU slope approach). Second, we used more than one relationship with wind speed to calculate oxygen flux; this allowed us to account for a variety of physical processes that affect dissolved oxygen concentrations, including breaking waves and bubble entrainment [27; see below]. We applied all of these windspeed relationships to both the BDO approach of Needoba et al. [26] and to our AOU slope approach.

For the first part of these calculations, DO and oxygen solubility (OS) were used to compute AOU as the difference between OS and DO for ten minute averages of high frequency measurements. AOU measures the cumulative effects of biological activity that have occurred in a water sample and is negative when oxygen is supersaturated, and positive when oxygen is undersaturated (due to consumption of DO). DO drawdown was calculated during nighttime (when incoming solar radiation was zero) based on regressions between AOU and time, which were tested for significance. Nighttime DO drawdown may differ significantly from daytime DO drawdown [28], meaning that nighttime CR rates may differ slightly from actual rates. This is an unavoidable limitation of using autonomously collected DO data to calculate CR.

We calculated gas exchange based on the air-sea concentration gradient and established parameterizations based on wind speed, where the diffusive flux (F) of oxygen equals the difference between the observed oxygen concentration of surface water and expected saturation oxygen concentration (which depends on temperature and salinity), multiplied by the gas transfer velocity (or coefficient of gas exchange; k_O2_) for oxygen at a given temperature (eq. 1) [29], [30], [31].
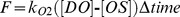
(eq.1)


Oxygen is transferred from the water to the atmosphere when F (umol cm^−2^) is positive, while oxygen is transferred from the atmosphere into the water when F is negative. Flux was calculated every ten minutes based on the measured wind speed, and was integrated over the dark period to obtain the total nighttime flux for each night. Nighttime integrated flux was subtracted from the entire nighttime AOU respiration estimate while each ten minute flux estimate was subtracted from each interval calculated following Needoba et al. [26].

k_O2_ was obtained by first computing k_660_ (cm hr^−1^) based on wind speed: this is the k value for CO_2_ at 20°C and has been measured empirically for various wind speeds in the ocean. There are multiple mathematical equations relating wind speed to k_660_ and we used six relationships to ultimately calculate oxygen flux and CR; these equations vary in the formulation of k as function of wind speed, especially whether relationships are linear [32], quadratic [30], [33], cubic [34], or power functions [32]. Needoba et al. [26] recommend using CR1, while CR2 and CR3 explicitly consider the higher *k* values resulting from breaking waves and bubble entrainment [27], [33], [34]. CR4-CR6 were specifically designed to capture lower wind speeds. We include all six approaches given the wide variation in environmental conditions across the Pacific and to provide broader context. All of the equations were originally derived using chemical tracers such as sulfur hexafluoride.
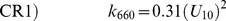
(eq.2)
[30]

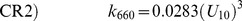
(eq.3)
[34]

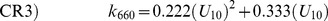
(eq.4)
[33]

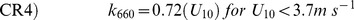
(eq.5)
[32]

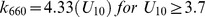


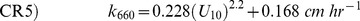
(eq.6)
[32]

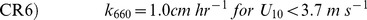
(eq.7)
[32]

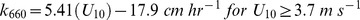



In all of these equations, wind speed (U_10_) is measured in m s^−1^ at 10 m above the water's surface whereas our data were collected at 1 m; wind speeds were therefore scaled to 10 m (eq. 8) [35]:
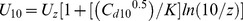
(eq.8)


Where C_d10_—the surface drag coefficient for wind above water at 10 m—is 1.3×10^−3^ [36], K refers to the von Karman constant of 0.41 [37], and z is the height above the sea surface where wind speed was measured. The Crusius & Wanninkhof [32] relationships were specifically developed for low wind speeds that may periodically occur over the ocean. In our data, wind speed was greater than 3.7 m s^−1^ 96.5% of the time.

Schmidt numbers (Sc) were used to covert from the modeled k_660_ value in equations 2–6 to a k_O2_ at each recorded surface temperature. A Schmidt number is a dimensionless number that characterizes fluid flow, is unique for each dissolved gas, and is defined as the kinematic viscosity of water divided by the diffusion coefficient of the gas at a given temperature. The Schmidt number for O_2_ in saltwater can be calculated with the following equation, in which T is temperature in Celsius (eq. 9) [30]:
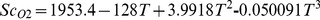
(eq.9)


The ratio of k values (for each CO_2_ and O_2_) equals the ratio of Schmidt numbers raised to negative n (eq. 10) [38]:
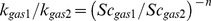
(eq.10)n depends on the processes that dominate gas transfer including the friction velocity and the mean square slope of the waves [39]. Based on the laboratory experiments of Jahne et al. [39], [40] we set n to 2/3 when U_10_<2 m s^−1^ and n at 1/2 when U_10_≥2 m s^−1^.

Computed oxygen fluxes were then divided by the mixed layer depth, and subtracted from the nighttime slopes of AOU versus time to obtain respiration rates. (Note that fluxes are defined relative to the atmosphere, and a negative flux represents transfer of oxygen into the ocean from the atmosphere.) Positive CR values indicate a higher rate of respiration while a ‘negative’ CR value indicates that DO increased during nighttime hours. Negative CR can be explained by daytime primary production exceeding and masking the amount of nighttime CR that occurred, with isopycnal mixing also potentially playing a role, as these measurements were only made at the surface, whereas photosynthesis and respiration likely varied with depth throughout the mixed layer.

### Data analysis

Two outliers (3-9-2012, and 12-31-2012) were removed from the California to Australia dataset due to extremely high calculated respiration values (>2 standard deviations from the mean). One outlier was removed from the California to Japan dataset (3-6-2012) due to extremely high chl *a* and turbidity values (>2 standard deviations from the means).

Relationships between respiration, latitude, temperature, chl *a*, and turbidity were explored using multiple regression analysis in R. Regression analysis was completed for all data, for each individual glider, and for latitudinal and longitudinal zones for the California to Australia dataset. These zones follow Longhurst's biogeographical zones [41], [42] and ranged from 20°N to 38°N (California Current province), 10°N to 20°N (North Pacific [Sub]Tropical Gyre province), 0°N to 10°N (North Pacific Equatorial Countercurrent province), 15°S to 0°S (Pacific Equatorial Divergence province), and 23°S to 15°S (South Pacific Subtropical Gyre province). The natural log was taken of chlorophyll and turbidity in order to correct slight non-linearity. Due to several slightly ‘negative’ values (see Discussion for an explanation), turbidity was offset by 1.38 so that the minimum value was 1 before transformation.

## Results

### Pacific-scale Patterns in CR

**Figure 2:**
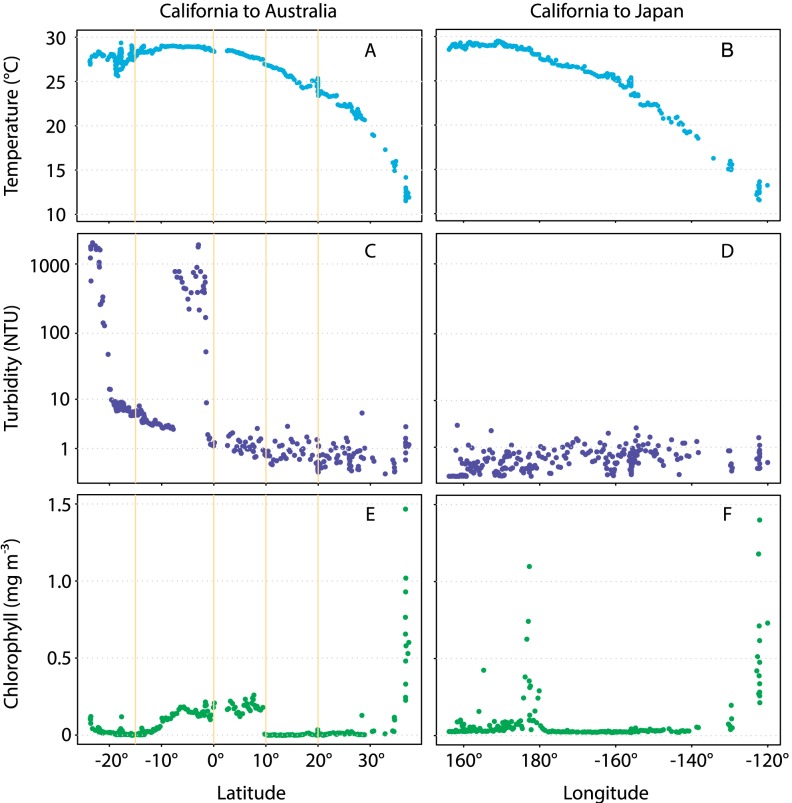
Environmental data collected by the gliders along the two transects. Panels depict temperature (°C) according to latitude for California to Australia (A) and according to longitude for California to Japan (B); turbidity (NTU) from California to Australia (C) and California to Japan (D); and Chlorophyll (RFU) from California to Australia (E) and California to Japan (F).

Data from autonomous gliders provide ocean-scale patterns in temperature, chlorophyll, turbidity, and respiration; all showed substantial variation across the Pacific Ocean. For the California to Australia transect, water temperature increased as latitude decreased, and was warmest just south of the equator (29.3°C) (Figs. 1, 2A and 2B). Water temperatures increased from the eastern to western Pacific along the transect from California to Japan. Chlorophyll concentrations were greatest off coastal California (above 30°N latitude) and were generally low in the open Pacific, with the exception of the elevated levels observed near the equator (Fig. 2E and 2F). Chlorophyll also increased near 18–19°N in the West Pacific, and turbidity was extremely variable throughout the Pacific (Fig. 2). Several of these variables were correlated with one another across the Pacific, along the different transects, or within different biogeographical zones (Table 1).

**Table 1:** r values for relationships among environmental datasets in different regions of the Pacific.

	Pacific Ocean	California to Japan	California to Australia	20 to 38°N	10 to 20°N	0 to 10°N	−15 to 0°S	−23 to −15°S
**Chlorophyll and temperature**	−0.418	−0.423	−0.410	−0.795	0.341	Not significant	0.592	Not significant
**Temperature and turbidity**	0.276	0.454	−0.288	Not significant	−0.270	Not significant	0.458	Not significant
**Chlorophyll and turbidity**	Not significant	Not significant	Not significant	Not significant	Not significant	Not significant	0.639	0.647

**Figure 3:**
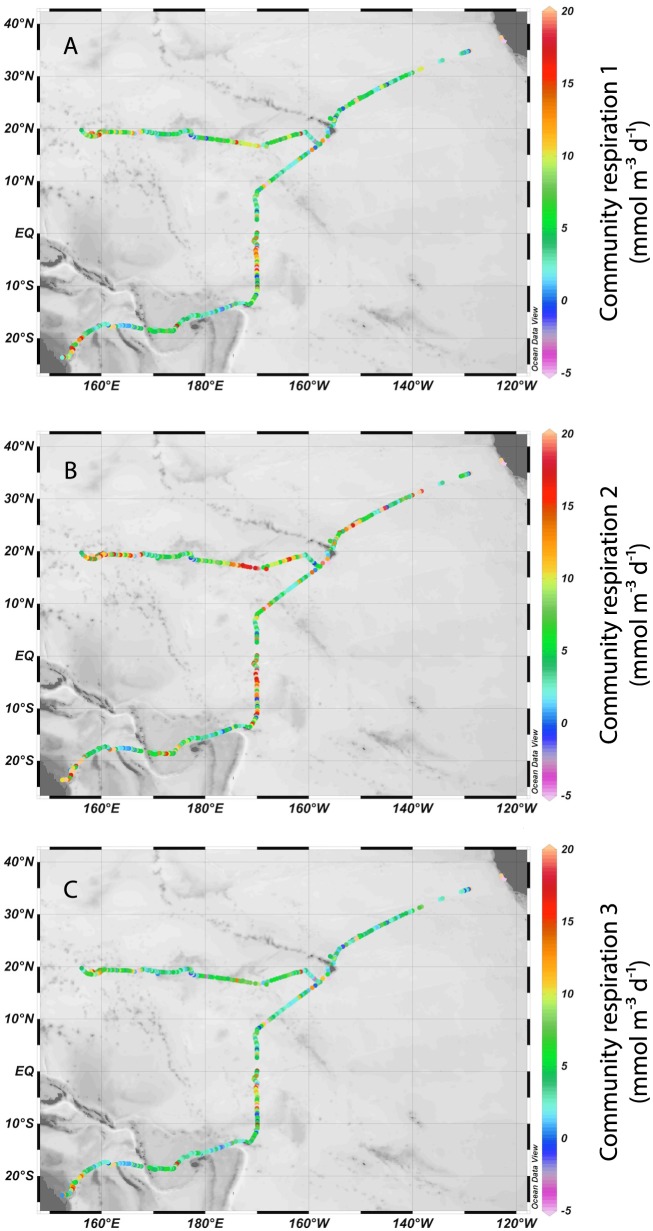
Computed community respiration rates along the two transects. Color shading shows (A) community respiration 1 (mmol m^−3^ d^−1^) (B) community respiration 2 (mmol m^−3^ d^−1^) and (C) community respiration 3 (mmol m^−3^ d^−1^) across the Pacific Ocean. 14 datapoints, 24 datapoints, and 15 datapoints exceed 20 mmol m^−3^ d^−1^and appear pink for CR1, CR2, and CR3, respectively; 19 datapoints, 15 datapoints, and 19 datapoints fall below −5 mmol m^−3^ d^−1^ and appear light purple for CR1, CR2, and CR3, respectively. Data displayed using Ocean Data View [59].

**Figure 4:**
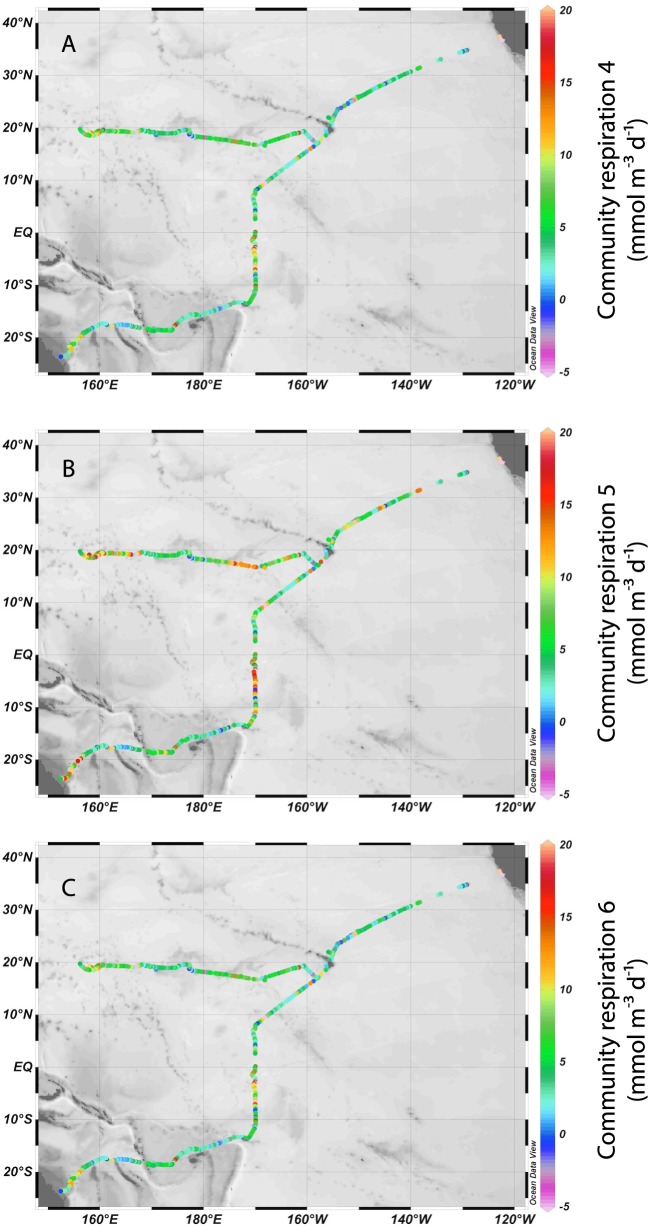
Computed community respiration rates along the two transects [58] **.** Color shading shows (A) community respiration 4 (mmol m^−3^ d^−1^) (B) community respiration 5 (mmol m^−3^ d^−1^) and (C) community respiration 6 (mmol m^−3^ d^−1^) across the Pacific Ocean. 14 datapoints, 16 datapoints, and 39 datapoints exceed 20 mmol m^−3^ d^−1^and appear pink for CR4, CR5, and CR6, respectively; 19 datapoints, 18 datapoints, and 14 datapoints fall below −5 mmol m^−3^ d^−1^ and appear light purple for CR4, CR5, and CR6, respectively. Data displayed using Ocean Data View [59].

In total, we generated 341 independent measurements of CR from California to Australia and 264 independent measurements from California to Japan. All of our computational approaches demonstrated that respiration rates were variable across the Pacific. The different windspeed-based approaches to calculate CR ashowed strong agreement, with r^2^ values ranging from 0.810–0.999 (all *P*<0.001) between different CR datasets within a given method for calculating respiration (AOU change over time, or summed changes in DO based on Needoba et al. [26]) (Tables 2 and 3). Independent of the windspeed-based flux estimates, the AOU and BDO approaches for estimating oxygen consumption were significantly correlated (*P*<0.001) for each transect, but the California to Japan dataset had an r^2^ value of 0.701 while the California to Australia dataset had an r^2^ value of 0.3. The BDO approach typically generated more instances of negative CR rates (Figure S1)—most likely due to summing many small changes in DO—and so our analysis is focused on the AOU slope approach. These six CR datasets varied slightly in terms of maximum, minimum, mean, and median values (Figs. 3 and 4) but were generally comparable: for example, CR1 rates ranged from −88.9 to 75.4 mmol m^−3^ d^−1^, CR2 ranged from −88.6 to 77.2 mmol m^−3^ d^−1^, CR3 ranged from −89.0 to 75.0 mmol m^−3^ d^−1^, CR4 ranged from −89.2 to 74.7 mmol m^−3^ d^−1^, CR5 ranged from −88.8 to 76.0 mmol m^−3^ d^−1^, and CR6 rates ranged from −89.1 to 74.9 mmol m^−3^ d^−1^ for the California to Australia dataset. We focus on CR2 because it considers breaking waves and bubble entrainment, the method used to obtain this relationship utilizes long-standing protocols, and it performs better statistically (see below). CR2 ranged from −120 to 84.4 mmol m^−3^ d^−1^ from California to Japan using our AOU slope approach (both for CR2; Fig. 3), while using Needoba et al. 's BDO approach [26], CR2 ranged from −58.9 to 61.9 mmol m^−3^ d^−1^ for the California to Australia dataset, and from −84.8 to 93.9 mmol m^−3^ d^−1^ for the California to Japan dataset.

**Table 2:** r^2^ for comparisons among different AOU approaches to calculating CR for the California to Australia (above the diagonal) and California to Japan (below the diagonal datasets).

	CR1	CR2	CR3	CR4	CR5	CR6	Raw
**CR1**		0.905	0.993	0.979	0.986	0.986	0.842
**CR2**	0.917		0.856	0.810	0.957	0.826	0.601
**CR3**	0.996	0.881		0.988	0.959	0.994	0.899
**CR4**	0.985	0.839	0.995		0.940	0.999	0.883
**CR5**	0.990	0.961	0.973	0.953		0.948	0.750
**CR6**	0.990	0.853	0.998	0.999	0.961		0.891
**Raw**	0.921	0.718	0.952	0.965	0.860	0.961	

P<0.001 in all instances.

**Table 3:** r^2^ for comparisons among different BDO approaches to calculating CR for the California to Australia (above the diagonal) and California to Japan (below the diagonal datasets).

	CR1	CR2	CR3	CR4	CR5	CR6	Raw
**CR1**		0.923	0.993	0.984	0.987	0.989	0.870
**CR2**	0.914		0.881	0.848	0.968	0.860	0.665
**CR3**	0.996	0.876		0.990	0.963	0.996	0.919
**CR4**	0.985	0.834	0.995		0.951	0.999	0.903
**CR5**	0.990	0.960	0.972	0.952		0.956	0.788
**CR6**	0.989	0.848	0.998	0.999	0.959		0.911
**Raw**	0.921	0.712	0.952	0.965	0.859	0.961	

P<0.001 in all instances.

The gliders traveled nearly identical paths from California to the Hawaiian Islands over slightly different time periods, yet they showed highly similar patterns in CR: both were variable and reached maximum and minimum values near the California Coast, and each was more consistent beyond 128°W. Several local maxima were recorded at various points within the northeastern portion of the North Pacific Gyre, particularly near 150°W. This included high CR rates measured at 148.7°W on 2012-02-09 and 156.2°W on 2012-03-13 for the California to Australia transect, and at 151°W on 2012-02-19 and 153.1°W 2012-03-09 for the California to Japan transect (for CR2). These local maxima were slightly greater for the California to Japan transect (39.7 mmol m^−3^ d^−1^ and 37.2 mmol m^−3^ d^−1^) than the California to Australia transect (35.3 mmol m^−3^ d^−1^ and 17.9 mmol m^−3^ d^−1^).

The east-to-west, California to Japan transect then crossed a large swath of the oligotrophic North Pacific Subtropical Gyre (NPSG), whereas the northeast-to-southwest, California to Australia transect crossed multiple ocean provinces (Figs. 3 and 4). We expected that the east-west transect would therefore show relatively low rates and little variation, but this was not the case: CR2 averaged 8.38 mmol m^−3^ d^−1^ with a standard deviation of 8.44 mmol m^−3^ d^−1^. Many of the high CR rates were observed within the NPSG—particularly in the central Pacific to the west of Hawaii—as well as closer to mainland Asia. The overall range of CR values was similar for the California to Australia transect, and also showed substantial spatial variability. CR above 20°N latitude was variable between days, latitude, and longitude with no obvious increasing or decreasing trends, but became less variable after 128°W longitude and remained below 10 mmol m^−3^ d^−1^ on all but 6 occasions after leaving the California Coast. CR rates were generally low from Hawaii to the equator, but a small peak in CR occurred near 10°N latitude. CR rates exhibited several obvious peaks below the equator and were consistently elevated from the equator to 10°S. CR2 averaged 6.49 mmol m^−3^ d^−1^ with a standard deviation of 11.0 mmol m^−3^ d^−1^ for the entire transect, but reached 26.2 mmol m^−3^ d^−1^ at 6.36°S latitude; CR was generally elevated within several degrees of latitude of this peak. CR again increased around 18–19°S, and the greatest values south of the equator were observed near the coast of Australia (46.5 mmol m^−3^ d^−1^ at 22.5°S latitude).

### Relationships to environmental data

We used several statistical approaches to analyze relationships between possible explanatory variables and CR. No approach to calculating CR was uniformly most significantly related to environmental data, however CR2 generally outperformed the other approaches for both datasets, and for both univariate and multiple linear regression by producing more significant relationships and stronger significant relationships. Most of our analysis therefore focuses on CR2, however all of the CR datasets were correlated and yielded similar results despite differences in their underlying assumptions. For these analyses, we examined both transects together as well as separately, and also analyzed the California to Australia dataset based on multiple latitudinal zones. This stems from the large expected and observed differences in oceanographic conditions across these regions, as well as the fact that datasets from different latitudes exhibit different relationships between temperature and CR (e.g., [15]).

Across the long oceanographic transects, CR2 varied significantly with turbidity from California to Australia (r^2^ = 0.085 and *P*<0.001), and increasing the number of possible explanatory variables in stepwise multiple linear regression did not increase the predictive strength of the model. For the California to Japan data, CR again varied most strongly with turbidity (r^2^ = 0.112, *P*<0.001); the most descriptive multiple regression model included chlorophyll and turbidity as explanatory variables (r^2^ = 0.167 and *P*<0.001) using CR2. When California to Australia data were assessed according to latitudinal zones, different explanatory variables were important in different areas. From north to south, no significant relationships were observed from 20°N to 38°N, 10°N to 20°N, and 0°N to 10°N, whereas chlorophyll was most significantly related to CR from 15°S to 0°S (r^2^ = 0.166, *P*<0.001 using CR2), and turbidity was the best predictor from 23°S to 15°S (r^2^ = 0.325, *P*<0.001 using CR2). Multiple linear regression did not yield any significant relationships north of the equator, and only increased the explanatory power of the model from 23°S to 15°S slightly, such that water temperature and turbidity explained 37.3% of the variation in CR (*P*<0.001).

**Figure 5:**
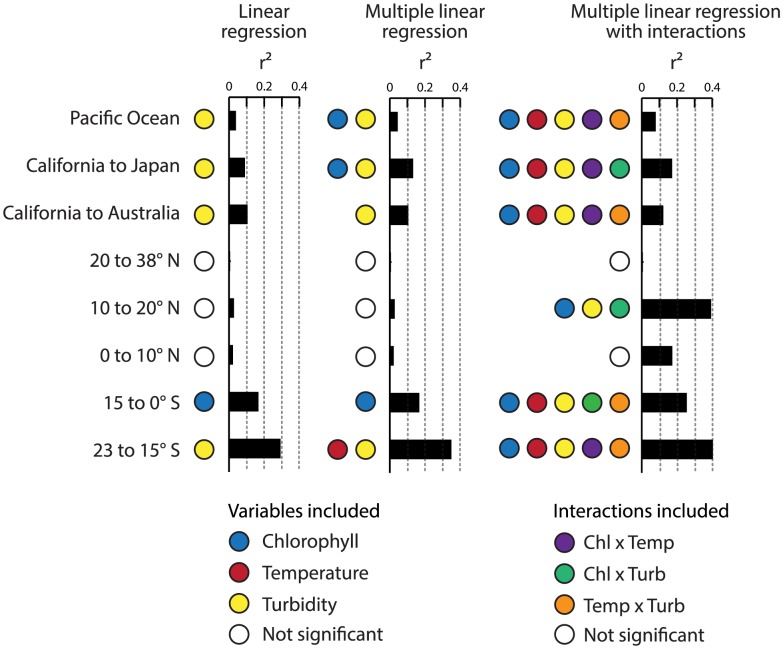
Regression statistics for best AIC derived relationships between AOU-slope-based CR2 data and environmental data. Bar graphs show regression r^2^ values for univariate linear regression, multiple linear regression, and multiple linear regression with interactions. Color-coded symbols next to the bar graphs indicate which variables or interactions yielded significant relationships; a white circle indicates that no significant relationships were found. These model statistics represent log-transformed turbidity and chlorophyll data.

Chlorophyll and turbidity were also log-transformed to fix slight non-linearity but produced highly similar results (Figure 5): CR2 had the most significant models; no significant relationships occurred north of the equator; chlorophyll was the best predictor from 15°S to 0°S (r^2^ = 0.165, *P*<0.001); and water temperature and turbidity were collectively the best predictors from 15°S to 23°S (r^2^ = 0.347, *P*<0.001). Allowing second or third order interactions to the log-transformed variable models for CR2 increased the explanatory power of all the models except from 20°N to 38°N (Fig. 5). For example, there were no significant relationships between CR and any of the variables between 10°N to 20°N, but adding an interaction between chlorophyll and turbidity resulted in a significant relationship (r^2^ = 0.391, *P*<0.001). Adding a third order interaction between water temperature, chlorophyll, and turbidity strengthened this relationship (r^2^ = 0.518, *P*<0.001).

All data were ultimately pooled together to analyze cross-Pacific patterns in respiration as a function of chlorophyll, turbidity, and temperature. Across the dataset, the strongest relationship was between the natural log of turbidity and CR2 (r^2^ = 0.036, *P*<0.001). Using multiple explanatory variables only incrementally increased the amount of variation explained, with the natural logs of chlorophyll and turbidity producing the best model (r^2^ = 0.043, *P*<0.01 for CR2). Allowing interactions between explanatory variables likewise incrementally increased the explanatory power of the models (e.g., r^2^ = 0.077, *P*<0.001 for CR2 allowing interactions between temperature, chlorophyll, and turbidity). A third order interaction term between water temperature, the natural log of chlorophyll, and the natural log of turbidity only explained 8.54% of the data (*P*<0.001 for CR2). We also used multivariate adaptive regression splines (MARS) [43] to model CR; this approach includes interactions among variables, and identifies regions of the dataset where different basis functions may be applied. The MARS model explained 7.5% of the variation in CR across the Pacific (*P*<0.001) as a function of temperature, chlorophyll, and turbidity.

For these pooled data, we found stronger relationships within the NPSG than across the entire Pacific. The NPSG is Earth's largest ecosystem [44], [45] and different regions of the NPSG were covered extensively by the two gliders. The natural log of turbidity was the most significant explanatory variable for 4 of the 6 methods of calculating CR (r^2^ = 0.110, *P*<0.001 for CR2), with water temperature being more strongly correlated with CR4 (r^2^ = 0.041, *P*<0.002) and CR6 (r^2^ = 0.033, *P*<0.005), from 10°N to 20°N in the NPSG. Including both of these variables in multiple regression (r^2^ = 0.202, *P*<0.001 for CR2) and their interactions (r^2^ = 0.245, *P*<0.001 for CR2) produced stronger relationships.

## Discussion

The CR rate data presented here are unique in both spatial coverage and the frequency with which measurements were taken, as no previous study has calculated CR continuously across an entire ocean basin. While CR has been measured across a few latitudinal and longitudinal transects, these consist largely of discrete measurements made at a limited number of locations [20], [23], [24]. Our data also provide a large number of measurements from the undersampled Pacific Ocean: in the most recent compilation of CR measurements, Regaudie-de-Gioux and Duarte [46] report 3854 measurements, with only 296 made in the Pacific Ocean (some of which are unpublished). We report 341 measurements from north to south, and 264 measurements from east to west, across the Pacific, more than tripling the total number of CR measurements made in Earth's largest ocean. Our data and findings are in broad agreement with the limited information available from the Pacific and other large-scale studies, display several interesting spatial patterns, and provide new insight into the environmental drivers of CR.

Most measurements of CR in the Pacific Ocean have been conducted at Station ALOHA (A Long Term Oligotrophic Habitat Assessment) of the Hawaii Ocean Time-series (HOT) program, and both gliders were in the vicinity of Station ALOHA for several days—approaching from the east and passing to the south (Figure 1). This allows for comparison of CR between the glider dataset and the extensive HOT dataset. In a year-long study at ALOHA from 2001–2002, Williams et al. [22] found that surface CR at station ALOHA ranged from 0.63 to 1.15 mmol O_2_ m^−3^ d^−1^; unpublished CMORE data from more recent HOT cruises show that surface respiration varies from 0.18 to 2.8 mmol O_2_ m^−3^ d^−1^ [46], [http://cmore.soest.hawaii.edu/cmoredata/Church/GPP_NCP_R_results_summary.xls]. These data illustrate the fact that even stratified, oligotrophic, open ocean sites exhibit order-of-magnitude variations in CR over time [22]. For the gliders, mean CR values ranged from 2.38-4.64 mmol m^−3^ d^−1^ near station ALOHA. These values overlap the upper end of CR rates from dark bottle incubations conducted at station ALOHA.

Glider-based CR data are also consistent with other datasets collected in the equatorial Pacific. Viviani et al. [23] measured CR, GPP, and NCP along a transect from 14.3°S, 169.2°W to Station ALOHA that lies to the west of our CA-Australia transect; they observed increased CR near the equator, with a maximum around 10°S. In our data, CR varied widely near the equator and in association with high chlorophyll and turbidity that are likely indicative of a phytoplankton bloom; CR appears to have responded by increasing between the equator and 10°S latitude. NCP and GPP also varied substantially over relatively short distances (∼50 km) along the equator in the Western Equatorial Pacific [47]. While Stanley et al. 's data were primarily collected along an east-west transect, and they did not report volumetric CR rates, they followed 170°W for ±2 degrees north and south of the equator. NCP decreased from north to south along 170°W, which could reflect decreased GPP, increased CR, or both. In our data, the glider *Benjamin* detected an increase in CR from north to south also while tracking 170°W, which is consistent with but not directly comparable to Stanley et al. [47].

As a whole, our datasets substantially increase the number of CR measurements available for the Pacific, and our approach may be useful in constraining the metabolic balance of the oligotrophic ocean [24], [48], [49], [50], [51], [52]. *In situ* studies of oxygen production and consumption tend to indicate that the open ocean is net autotrophic while *in vitro* studies indicate net heterotrophy (see [24]). Setting aside issues regarding the depth of integration, the discrepancies most likely have to do with an underestimation/overestimation of photosynthesis rather than mistakes calculating respiration [24], [53]. This view is supported by comparisons of in vitro and *in situ* data from HOT, which found general agreement between respiratory rates for the two approaches [22], [53], [54]. While most of the global CR database is comprised of light-dark bottle measurements [19], [46], our data represent a large collection of *in situ* measurements that provide a useful point of comparison. Clearly some uncertainty lies in gas exchange, which leads to variations in absolute magnitudes among our CR datasets—however the variation in gas exchange as a function of windspeed (typical *r^2^* values of 0.8±0.1) is a long-running issue without clear resolution (reviewed by [27]). Regardless of the approach we used to calculate CR, the majority of the data fell within observations reported elsewhere in the ocean [19], with >90% falling within the range of surface CR rates reported in Regaudie-de-Gioux and Duarte's 2013 dataset [46]. The remaining values are almost entirely ‘negative’ CR rates. Our approach could therefore be adapted to daytime increases in oxygen and used to calculate patterns in surface NCP across the Pacific.

Based on these data we observed several large-scale patterns in CR across the Pacific Ocean, including high CR rates near the equator and the coast of Australia, and surprising variation in CR within the NPSG. While rates were low throughout much of the NPSG, high CR rates may occur at least transiently in portions of the NPSG west of Hawaii. CR was also highly variable in the South Pacific, exhibiting a wide range of values and sharp changes within zones. These data represent the first extensive series of measurements made in the highly undersampled South Pacific Subtropical Gyre [19], [42], [46] and indicate that large gradients in CR may occur in this ocean. For the most part, these variations were not explained by coincident turbidity, temperature, and chlorophyll measurements made by the gliders. Previous work in the Atlantic Ocean has shown significant relationships with beam attenuation [55], which is consistent with the presence of particles and aggregates in the water column that may be subsequently remineralized and respired. Turbidity was frequently related with CR, including significant relationships across the whole dataset and for each transect. However, there are two main issues with the turbidity data collected by the PacX gliders. First, there were many low, but negative, turbidity values that likely reflect sensor drift over the extended deployment at sea (Fig. 2C). Second, microbubbles can potentially interfere with the turbidity sensor: as outlined by Villareal and Wilson [56], this likely explains the highly elevated turbidity values observed south of 18°S, as the glider *Benjamin* traversed Tropical Storm Freda and may have had bubbles entrained in the sensor. While the significant relationships observed in other biogeographic regions and for the other glider correspond with much lower turbidity values, we cannot exclude the possibility that these stem from artifacts present in the data.

Temperature was generally weakly correlated with CR—if at all—and was only a significant predictor in multiple linear regression. The slopes of these relationships were variable, ranging from negative to positive. This contrasts with some previous work [18], [19], but supports the idea that temperature has mixed effects on CR in different regions of the ocean, and specifically the idea that temperature effects are weak outside of high latitude regions [15]. Kirchman et al. [15] convincingly argue that temperature is not a strong regulating factor in other parts of the ocean because the availability of organic C substrates and nutrients is more important. This likely applies to much of our dataset, as outside of the region that extends from California to 20°N, temperatures fell between a relatively narrow range of 23–30°C (representing >84% of the temperature data). No significant relationship between CR and temperature was observed from 20°–38°N—despite a wider range of temperatures—but no significant relationships were observed between CR and any of the *in situ* measurements in this region. This is due to the localized hotpots in CR observed near 150°W and 25°N, which are not associated with distinct changes in turbidity, temperature, or chlorophyll.

Compilations of CR datasets have also shown that CR can be positively correlated with chlorophyll concentrations [18], [19], [46]. Production ultimately sets the pace for CR rates, and it seems likely that coupling between CR and either the biomass (chlorophyll) or photosynthetic activity (GPP) of phytoplankton would co-vary across the ocean and through time. For example, several studies have found that chlorophyll consistently explains ca. 30% of the variance in CR—whether in estuaries, throughout the Atlantic Ocean, or across oceans [19], [55], [57], [58]. However, Regaudie-de-Gioux and Duarte [46] found that both net community metabolism and CR were each only weakly correlated with chlorophyll while 30% of the variability in GPP was explained by chlorophyll. Robinson and Williams [19] showed that, overall, CR is not strongly correlated with chlorophyll concentrations, while stronger relationships are observed in regions of the ocean with sharp chlorophyll and CR gradients. Our data from the region south of the equator in the Pacific are consistent with this: high CR rates observed south of the equator correspond with high chlorophyll concentrations (Fig. 2E). These latter data capture a phytoplankton bloom associated with equatorial upwelling, and CR was significantly related to chlorophyll in the latitudinal band just south of the equator for all CR datasets (Fig. 5). An increase in CR near the equator appears to be a general feature in the Atlantic Ocean [20], [55], [57] that was also observed by Viviani et al. [23] in Pacific Ocean, and our data extend these observations. These variations in CR are likely driven by upwelling in the equatorial oceans—either by directly affecting CR through nutrient supply, or by fueling GPP and increasing the supply of organic C to CR.

We also observed high CR rates where chlorophyll showed little change—especially high CR rates in the oligotrophic NPSG west of Hawaii, and low CR rates where chlorophyll was elevated near the International Date Line. Chlorophyll and CR were in fact inversely related along the California to Japan transect. This reflects decoupling between phytoplankton biomass and CR and is in line with the intensive measurements made by Williams et al. [22] at Station ALOHA: they argue that primary production occurs in intermittent bursts—which they may not have detected even with comprehensive sampling—whereas CR is less variable, and more integrative, over time. Our cross-Pacific data suggest that another distinguishing feature of different oceanic provinces may be differences in the coupling between primary production and CR, with strong coupling in productive provinces, and decoupling in less productive regions. Sampling discrete stations may obscure this pattern, because our data demonstrate that isolated hot spots or moments of CR occur in oligotrophic regions. This finding has implications for understanding carbon and metabolic balance in the sea, as it reinforces the importance of integrating CR over time and depth, but also lateral space.

The net effects of ocean warming and other forms of global change on CR will ultimately depend on a complex series of responses among communities of phytoplankton and heterotrophic microbes in different ocean provinces. For the first time, we provide basin-scale measurements of CR conducted at high spatial and temporal resolution. Like previous CR datasets, our data capture patterns over limited periods of time, and temporal variation may be intertwined with spatial differences. Unlike primary production, CR has not been measured regularly or systematically and cannot be easily sensed from space. Instead, use of spatially-distributed, high-quality, *in situ* biogeochemical measurements made at high frequency by autonomous gliders, floats, and moorings seems a promising approach for regular measurements of CR across the ocean. Our work provides CR data that are consistent in magnitude and pattern with previous data, demonstrating the feasibility of using autonomous platforms to measure CR over large scales. Such data allow us to identify broad biogeographic and biogeochemical patterns that are otherwise undetectable by isolated measurements. This reveals a more nuanced view of the environmental controls on CR, highlighting weak temperature effects in warm waters of the Pacific, and the coupling and decoupling between phytoplankton biomass and CR in different regions. Of particular relevance are hot spots and moments of CR, which may affect carbon flux estimates and represent interesting oceanographic and ecological phenomena. The novel dataset and approach provides hundreds of new measurements from the under-sampled Pacific, yielding new insight into variation in CR across the world's largest ocean.

## Supporting Information

Figure S1 **BDO computed community respiration rates along the two transects.** Color shading shows (A) community respiration 1 (mmol m^−3^ d^−1^) (B) community respiration 2 (mmol m^−3^ d^−1^) (C) community respiration 3 (mmol m^−3^ d^−1^) (D) community respiration 4 (mmol m^−3^ d^−1^) (E) community respiration 5 (mmol m^−3^ d^−1^) and (F) community respiration 6 (mmol m^−3^ d^−1^) across the Pacific Ocean. Data displayed using Ocean Data View [59].
(TIF)
