# Supplementary figures and images for: Ocean-Scale Patterns in Community Respiration Rates along Continuous Transects across the Pacific Ocean
Source: PLoS One. 2014 Jul 21;9(7):e99821. doi: 10.1371/journal.pone.0099821 (PMC4105538; doi:10.1371/journal.pone.0099821)

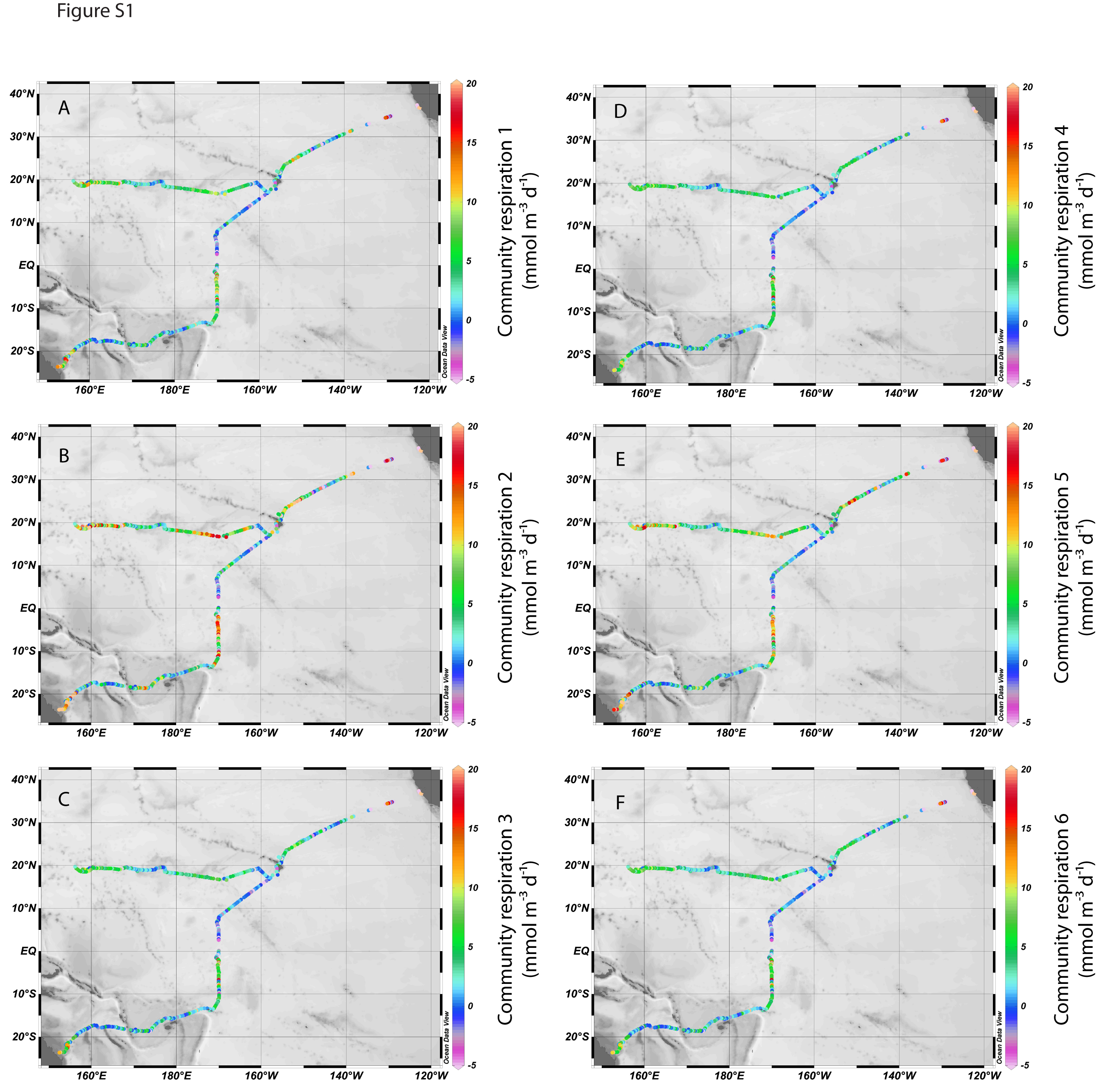

Supplement: Figure S1 — BDO computed community respiration rates along the two transects. Color shading shows (A) community respiration 1 (mmol m−3 d−1) (B) community respiration 2 (mmol m−3 d−1) (C) community respiration 3 (mmol m−3 d−1) (D) community respiration 4 (mmol m−3 d−1) (E) community respiration 5 (mmol m−3 d−1) and (F) community respiration 6 (mmol m−3 d−1) across the Pacific Ocean. Data displayed using Ocean Data View [59]. (TIF) [file pone.0099821.s001.tif]
